# Natural Nrf2 Inhibitors: A Review of Their Potential for Cancer Treatment

**DOI:** 10.7150/ijbs.82401

**Published:** 2023-06-04

**Authors:** Juan Zhang, Hong-Xi Xu, Jia-Qian Zhu, Yao-Xing Dou, Yan-Fang Xian, Zhi-Xiu Lin

**Affiliations:** 1 School of Chinese Medicine, Faculty of Medicine, The Chinese University of Hong Kong, Hong Kong SAR, China.; 2 School of Pharmacy, Shanghai University of Traditional Chinese Medicine, Shanghai 201203, China.; 3 The Second Affiliated Hospital of Guangzhou University of Chinese Medicine, Guangzhou, China.; 4 Hong Kong Institute of Integrative Medicine, Faculty of Medicine, The Chinese University of Hong Kong, Hong Kong SAR, China.; 5 Institute of Chinese Medicine, The Chinese University of Hong Kong, Hong Kong SAR, China.

**Keywords:** Natural products, Nrf2 inhibitors, Brusatol, Cancer, Molecular target

## Abstract

Nuclear factor erythroid 2-related factor 2 (Nrf2), a transcription factor that regulates redox homeostasis, plays a pivotal role in several cellular processes such as cell proliferation and survival, and has been found to be aberrantly activated in many cancers. As one of the key oncogenes, Nrf2 represents an important therapeutic target for cancer treatment. Research has unraveled the main mechanisms underlying the Nrf2 pathway regulation and the role of Nrf2 in promoting tumorigenesis. Many efforts have been made to develop potent Nrf2 inhibitors, and several clinical trials are being conducted on some of these inhibitors. Natural products are well-recognized as a valuable source for development of novel therapeutics for cancer. So far, a number of natural compounds have been identified as Nrf2 inhibitors, such as apigenin, luteolin, and quassinoids compounds including brusatol and brucein D. These Nrf2 inhibitors have been found to mediate an oxidant response and display therapeutic effects in different types of human cancers. In this article, we reviewed the structure and function of the Nrf2/Keap1 system and the development of natural Nrf2 inhibitors with an emphasis on their biological function on cancer. The current status regarding the Nrf2 as a potential therapeutic target for cancer treatment was also summarized. It is hoped that this review will stimulate research on naturally occurring Nrf2 inhibitors as therapeutic candidates for cancer treatment.

## Introduction

Cancer is a condition in which the cells of an organism multiply in an uncontrollable manner. Currently, over 200 different types of cancer have been identified in humans 1, and they present a leading cause of death and a significant hindrance to life expectancy worldwide. In 2020 alone, 19,292,789 new cancer cases and 9,985,133 cancer deaths were reported globally, equating to around 27,356 deaths per day 2. Carcinogenesis is a multistep process involving multiple genetic alterations which are necessary for a normal cell to develop into a tumor cell. This process is typically categorized into three stages: initiation, promotion and progression, and is characterized by various biological hallmarks 3. These hallmarks include cell transformation, genome instability, hyperproliferation, immortalization, angiogenesis, epithelial-mesenchymal transition (EMT) and metastasis. Many of these processes are influenced by intracellular reactive oxygen species (ROS) 4-6. The redox signaling process begins with the generation of ROS, which subsequently plays a role in signal transduction pathways involved in every step of carcinogenesis 7, 8. A high level of the oxidative stress, usually induced by ROS, is a significant characteristics of cancer cells. On the other hand, genes and signal transduction pathways commonly found in multiple cancer hallmarks are considered promising therapeutic targets.

Nrf2, also known as NFE2L2, is considered as the leading transcription factor controlling cell defense responses against various forms of cellular stresses, including oxidative stress 9. The stability of Nrf2 is subtly regulated by KELCH-like ECH-associated protein 1 (Keap1), which facilitates its degradation through ubiquitin-proteasome pathway in the absence of oxidative stress 10-12. As the primary stress regulator of cells, Nrf2 is intimately involved in tumor formation, progression and metastasis 13. Notably, the persistent and aberrant activation of Nrf2, which is a frequent occurrence in many tumors, provides cancer cells with a selective advantage, leading to tumor progression, metastasis, therapy resistance, and poor prognosis 14, 15. Over the past decades, Nrf2 has emerged as an auspicious target for cancer treatment, and numerous efforts have been made to identify therapeutic strategies that can disrupt its pro-oncogenic role 14. Inhibiting Nrf2 activation is an attractive and promising approach for cancer prevention, treatment and drug sensitization.

Natural products and Chinese herbal medicine (CHM) are known to possess various pharmacological or biological activities, some of which are beneficial for cancer therapy. Moreover, they are a valuable source of bioactive molecules that can be used for the development of pharmaceutical drugs 16. Several studies have demonstrated that natural products have the potential to counteract oxidative stress by modulating the Nrf2 pathway 17-23. In addition, natural compounds such as apigenin, luteolin, chrysin and brusatol have been shown to be potent Nrf2 inhibitors. These findings suggest that natural Nrf2 inhibitors could be utilized as chemopreventive and chemotherapeutic agents, as well as tumor sensitizers for conventional radiotherapy and chemotherapy. This review provides an overview of the major naturally occurring Nrf2 inhibitors and their underlying molecular mechanisms for Nrf2 regulation. It highlights the potential of these compounds in the treatment of human cancers. We hope this review will facilitate new ideas in the development of Nrf2 inhibitors as novel anticancer drugs.

## Nrf2 and Its Biological Functions and Regulation

Nrf2 is a 605 amino acid protein with a molecular weight of 68 kDa and encoded by NFE2L2 gene belonging to a transcription factor subfamily with the cnc ('cap'n'collar') structure. It contains a basic region‐leucine zipper DNA binding domain (b-Zip). So far, six members of this family have been identified: NF‐F2, Nrf1, Nrf2, Nrf3, Bach1 and Bach2. Nrf2 is considered to be the primary regulator in oxidative stress 24. It exhibits seven highly conserved Nrf2-ECH homology (Neh) domains designated as Neh1-7, respectively, which are important for Nrf2 functions 25, 26. Among them, Neh1 contains a b-Zip domain for DNA and small musculoaponeurotic fibrosarcoma proteins (sMafs) binding. The Neh2 domain mediates the interaction with the negative regulator Keap1 within specific binding sites known as ETG and DLG motifs. Neh3-5 are required for target genes transactivation and functional interaction with several modulators, while the Neh6 domain contains a serine-rich region and a degron that is involved in the degradation of Nrf2 in oxidative stressed cells. Finally, Neh7 domain can interact with an Nrf2 repressor called “retinoic X receptor α” which inhibits NEF2L2 target gene transcription 26, 27. Keap1 is a component of the Cullin 3 (CUL3)-based E3 ubiquitin ligase complex and controls the stability and accumulation of Nrf2. The Keap1 DC domain directly binds to Nrf2 through DLG and ETGE motifs within the N-terminal Neh2 domain of Nrf2 28. This two-site binding of Nrf2 and Keap1 has been shown to be the molecular basis of electrophile-induced Nrf2 accumulation 29. Inactivation of Keap1 strongly induces Nrf2, and this phenomenon is often observed in cancer cells, enabling them to acquire malignancy by perverting Nrf2 activity 15. Apart from Keap1-dependent mechanism, emerging evidence has revealed that Nrf2 can also be regulated through various mechanisms independent of Keap1 30.

Stabilized Nrf2 translocates into the nucleus and forms a heterodimer with a sMaf transcription factor 31. The Nrf2-sMaf heterodimer binds to antioxidant-responsive element (ARE) and induces transcription of numerous cytoprotective genes 15, 32. The target genes regulated by Nrf2/ARE include phase I, II detoxification enzymes and phase III drug transporters, as well as some other transcription factors 23. Briefly, phase I enzymes oxidize drugs or xenobiotics, while phase II enzymes conjugate products of phase I reactions, and phase III enzymes transport the final metabolites out of cells, cooperating to exert a cytoprotective function 27. So far, more than 200 Nrf2 target genes have been identified in humans 33. Most of them encode for metabolic enzymes that detoxify electrophiles (phase I/II/III drug metabolism) or scavenge ROS to restore the intracellular redox homeostasis and minimize the oxidative damage 34-36. Furthermore, accumulating evidence indicates that Nrf2 can also regulate other biological processes with physio-pathological relevance in human diseases (e.g., tumors), such as proliferation, apoptosis, mitochondrial function or biogenesis, inflammation and several metabolic pathways involved in pentose phosphate metabolism and fatty acid pathway. These biological processes are essential contributors to the maintenance of cellular redox and normal cell proliferation 14, 37, 38.

## Nrf2 Activation and Its Role in Cancer Development

**Figure 1:**
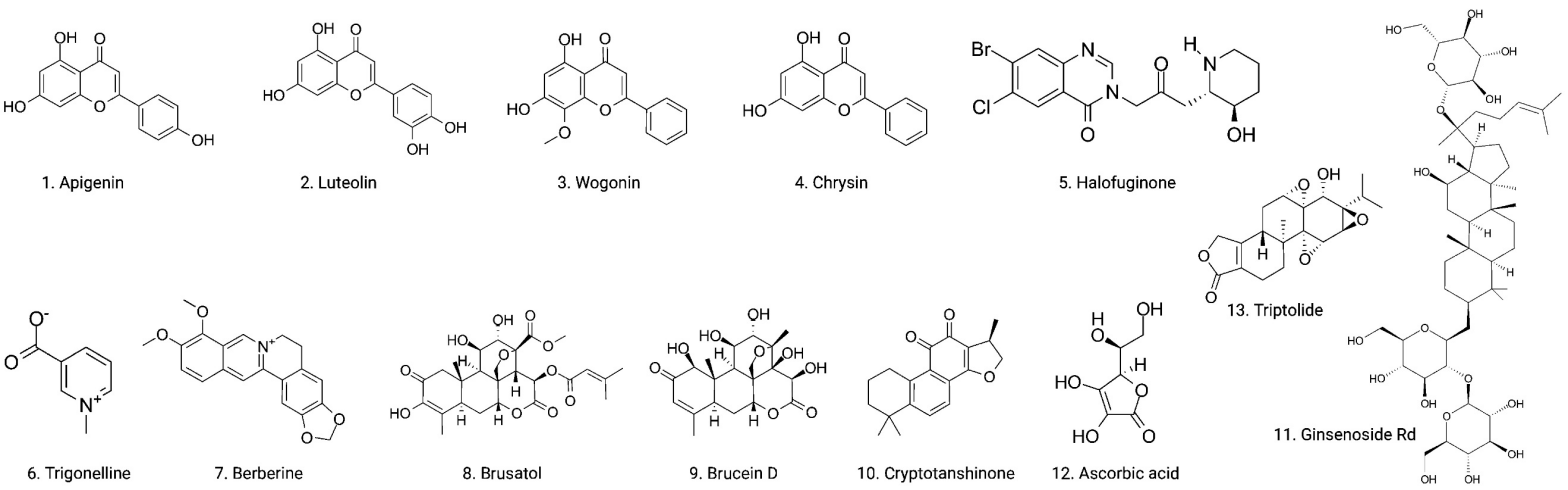
Chemical structures of naturally occurring Nrf2 inhibitors.

Many studies have amply demonstrated the physiological relationship among Nrf2, cellular redox homeostasis, and tumorigenesis 27, 39. Redox status imbalance commonly occurs in cancer 40. Tumor cells exhibit permanent high ROS levels owing to the oncogene activation, hypoxia, mitochondrial and/or peroxisomal dysfunction, increased metabolic rates, as well as anchorage-independent growth 41. In this context, Nrf2 plays a crucial role by acting as a major regulator of the antioxidant response. Furthermore, studies have shown that persistent activation of Nrf2 has been frequently observed in many types of human cancers, including lung, breast, esophagus, ovarian, head and neck, skin and renal cancers 42-44. Nrf2 activation can contribute to tumor development and progression by promoting cell survival, proliferation, and resistance to chemotherapy and radiation therapy (Figure 1) 45. Moreover, overexpression of Nrf2 has been linked to poor prognosis and resistance to chemotherapy in several types of cancer 46.

Several mechanisms have been proposed to explain how Nrf2 activation contributes to cancer development. Normally, Nrf2 is degraded through a Keap1-Cul3-Roc1-dependent mechanism. However, in human cancer, somatic mutations occur in Nrf2, altering amino acids in the DLG or ETGE motifs, resulting in the accumulation of Nrf2. Mutant Nrf2 cells display constitutive induction of cytoprotective enzymes and drug efflux pumps that are insensitive to Keap1-mediated regulation 47. Moreover, Nrf2 activation can increase the anti-apoptotic gene expression and promote carcinogen metabolism, leading to cell survival and increased DNA damage and mutation rates, which can further promote cancer development 48.

Overall, while Nrf2 activation is important for cellular homeostasis and protection against oxidative stress, it can also contribute to cancer development and progression. Therefore, targeting Nrf2 signaling may represent a promising therapeutic approach for cancer treatment, e.g. temporally inhibiting Nrf2-dependent cytoprotection using Nrf2 inhibitors can be utilized to enhance the responses to anticancer drugs 49.

## Natural Compounds as Nrf2 Inhibitors in Cancer

**Figure 2:**
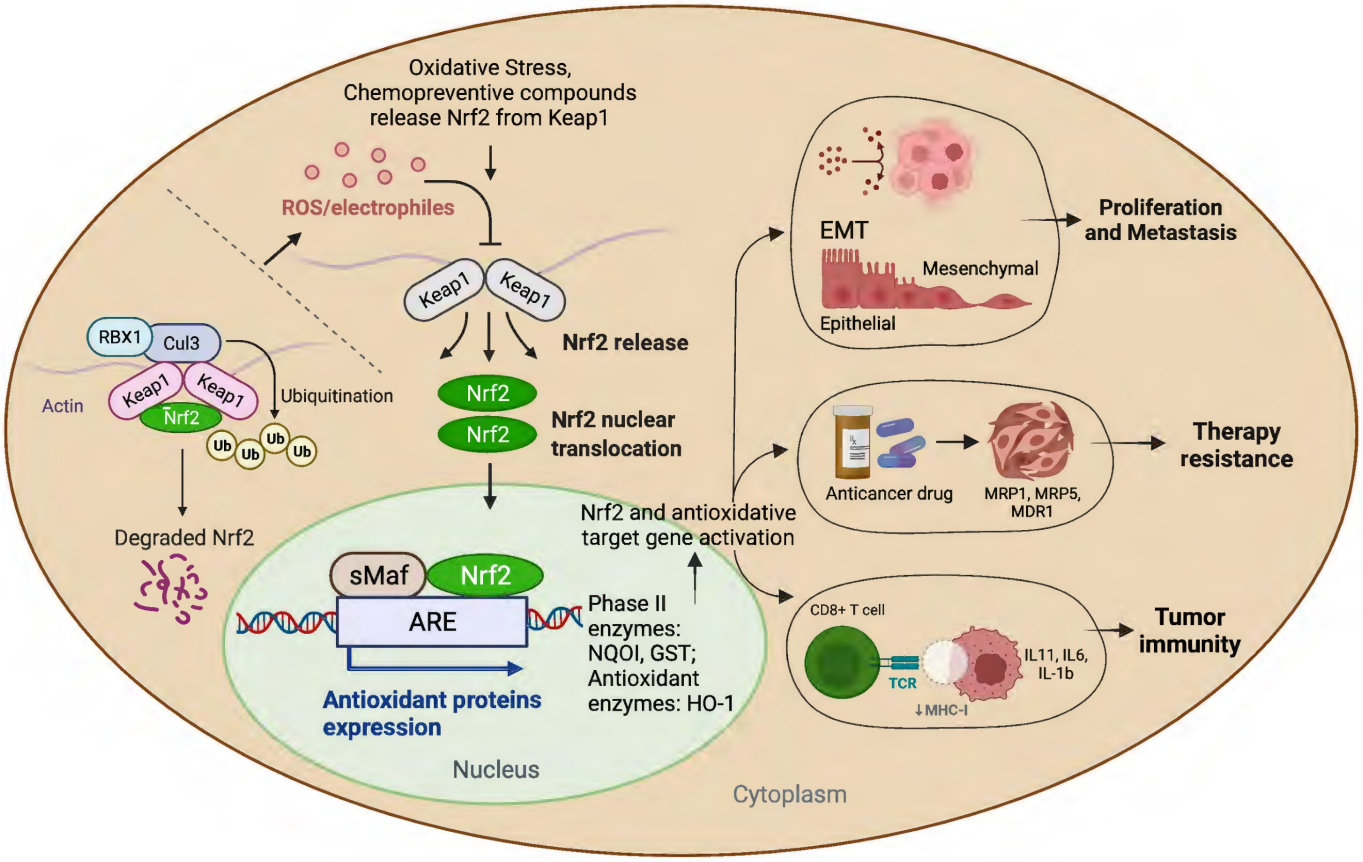
Schematic presentation of the role of Nrf2 in cancer progression.

**Figure 3:**
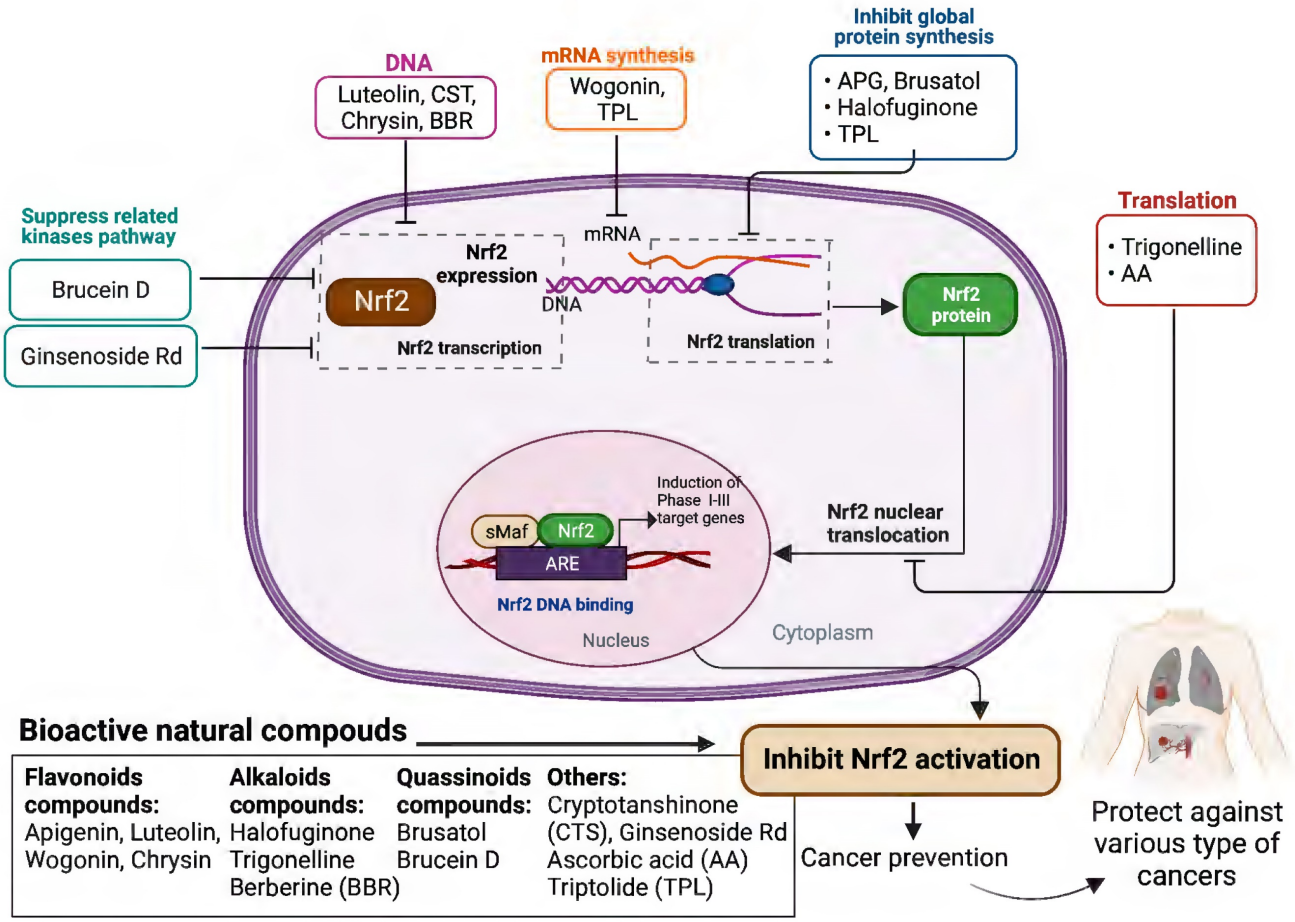
Schematic presentation of the molecular mechanisms underlying the cancer prevention and treatment of natural compounds via inhibiting Nrf2 pathway.

Based on the evidence concerning the role of Nrf2 in tumor cells, increasing number of natural products have been found to exert chemopreventive and therapeutic properties against different cancers via targeting Nrf2. These Nrf2 inhibitors include natural compounds extracted from plants, such as flavonoids, alkaloids and quassinoids. Moreover, some coffee extracts and vitamins have also been identified as Nrf2 inhibitors, including trigonelline and ascorbic acid (AA). We summarized the naturally occurring Nrf2 inhibitors below and discussed their biological significance related to cancer treatment in this section (**Figure 2-3** & **Table 1**).

### Flavonoids compounds

Flavonoids, a diverse group of natural polyphenolic compounds commonly occurring in plants, have been found to have strong anti-proliferative activity against many types of cancer and sensitize cancer cells to anticancer agents 50, 51. Several flavonoidal compounds, including apigenin, luteolin, wogonin and chrysin, have been reported to be Nrf2 inhibitors that can effectively reverse drug resistance.

#### Apigenin

Apigenin (4',5,7-trihydroxyflavone, APG) is a naturally occurring flavonoid widely present in fruits, vegetables and herbs 52. It has diverse biological activities such as anti-inflammatory, antioxidant and anti-carcinogenic properties. Studies have shown that APG as a potent Nrf2 inhibitor significantly sensitizes doxorubicin-resistant hepatocellular carcinoma (HCC) BEL-7402 (BEL-7402/ADM) cells to doxorubicin, increases intracellular concentration of ADM and suppresses tumor growth in a mouse xenograft model. Mechanistically, APG dramatically reduced the expressions of Nrf2 and its downstream genes through downregulating phosphatidylinositol-3 kinase (PI3K)/Akt pathway, leading to a reversal of drug-resistant phenotype 53.

#### Luteolin

Luteolin (3',4',5,7-tetrahydroxyflavone) is a polyphenolic flavonoid found in high concentrations in celery, parsley, green pepper, perilla leaf and chamomile tea 54. It has been shown to exhibit multiple pharmacological effects, such as antioxidative, anti-inflammatory, autophagy-regulatory, apoptotic and antitumor effects 54. Luteolin was found to be a potent Nrf2 inhibitor using a cell-based ARE-reporter assay. Data indicated that luteolin dramatically sensitized A549 cells to the anticancer drugs oxaliplatin, bleomycin, and doxorubicin, suggesting its potential application as a natural sensitizer in chemotherapy 55. Studies have suggested that luteolin could sensitize oxaliplatin-resistant colorectal cancer (CRC) cells to chemotherapeutic drugs through inhibition of the Nrf2 pathway 56. Studies have also demonstrated that luteolin could suppress the sphere formation of breast cancer stem cells (CSCs) and further enhance the chemosensitivity of anticancer drug taxol through downregulating Nrf2 expression via inhibiting the antioxidant genes HO-1 and Cripto-1, which have been known to contribute to CSC features. These findings indicate that luteolin may be a potential therapeutic agent for the cancer stemness-targeted breast cancer treatment 57.

#### Wogonin

Wogonin (5,7-dihydroxy-8-methoxyflavone), one of the major flavonoids isolated from the root of *Scutellaria baicalensis*, is a promising anticancer candidate owing to its anti-proliferative, apoptotic, cell migration-inhibitory, angiogenesis-inhibitory and differentiation-inducing activities 58. Studies have shown that wogonin could effectively combat chemoresistance in the Adriamycin (ADR)-induced resistant human chronic myelogenous leukemia (CML) K562/A02 and potentiated the inhibitory effect of ADR on leukemia *in vivo* through inhibiting Nrf2 signaling by Stat3/NF-kB inactivation 59. Similarly, other studies also reported that wogonin had strong reversal potency in the ADR-induced resistant human CML K562/A02 cells through inhibition of MRP1, decrease of the binding ability of Nrf2 to ARE and modulation of Nrf2 through reduction of Nrf2 mRNA in transcriptional processes 60. These studies strongly suggest that being a Nrf2 inhibitor wogonin holds good potential as an efficient natural sensitizer for anti-neoplastic resistant human myelogenous leukemia.

#### Chrysin

Chrysin (5,7-dihydroxyflavone), a natural flavonoid, is derived from many plant extracts such as blue passionflower, propolis and honey, all of which are widely used as herbal medicine in China. Chrysin has been demonstrated to possess multiple bioactivities such as antioxidant, anti-inflammatory, antibacterial and antitumor actions through experiments using a variety of human cancer cell lines 61. Chrysin could inhibit the cell proliferation, migration and invasion capacity in glioblastoma multiforme and inhibit tumor growth in U87 xenografts through inhibiting nuclear localization of Nrf2 and suppressing the expression of hemeoxygenase-1 (HO-1) and NAD(P)H quinine oxidoreductase-1 62. In doxorubicin resistant HCC BEL-7402/ADM cells, chrysin could significantly enhance their sensitivity to doxorubicin via inhibiting Nrf2 expression and downregulating the downstream genes such as HO-1, AKR1B10 and MRP5 63. These results indicate the effectiveness of using chrysin as an adjuvant sensitizer to combat chemoresistance.

### Alkaloid compounds

Alkaloids are a highly diverse group of compounds containing cyclic structures with at least one basic nitrogen atom being incorporated within, and they are important chemical compounds that serve as a rich reservoir for drug discovery 64. Numerous alkaloids have been screened from medicinal plants and herbs for their anticancer effects 65. Among them, febrifugine derivative halofuginone, trigonelline and berberine are well known as Nrf2 inhibitors and possess potent chemotherapeutic actions.

#### Halofuginone

Febrifugine, a naturally occurring alkaloid, is the active ingredient found in the root of *Dichroa febrifuga Lour* (Blue Evergreen Hydrangea), and has been used as a remedy to treat malaria, cancer, fibrosis, and inflammatory diseases 66. Febrifugine was identified as a potential Nrf2 inhibitor through a high-throughput chemical screening programme using lung adenocarcinoma A549 cells 67. Halofuginone is a less-toxic febrifugine-derivative that has been used as an antibiotic for animals 68. Halofuginone also exhibited Nrf2 inhibitory activity in a dose-dependent manner, and a study showed that the Nrf2-addicted cancer cells were more susceptible to its treatment than the normal epithelial cells. For instance, the IC_50_ values of halofuginone in normal epithelial cell lines BEAS-2B and NCC16-P11were 436.3 and 442.7 nM, respectively, while the IC_50_ values for the Nrf2-addicted cancer cells KYSE70 and A549 were 114.6 and 58.9 nM, respectively. It was also shown to enhance the anticancer effects of cisplatin *in vivo* 69. Taken together, halofuginone enhanced the chemosensitivity of cancer cells via suppressing Nrf2 activation. The results provide preclinical proof-of-concept evidence for halofuginone as a Nrf2 inhibitor which could be developed into therapeutic agent for chemoresistance.

#### Trigonelline

Coffee extracts were found to be modulators of Nrf2 nuclear translocation and ARE-dependent gene expression 70. Trigonelline, a coffee alkaloid that is abundantly present in coffee beans and many plants, efficiently decreased the basal and tBHQ-induced Nrf2 activity in several pancreatic carcinoma cell lines. In addition to inhibition of Nrf2, trigonelline also prevented the Nrf2-dependent expression of proteasomal genes. The sensitivity of pancreatic cancer cell lines to anticancer drugs, and tumor necrosis factor-related apoptosis inducing ligand (TRAIL)-induced apoptosis was potentiated by trigonelline 71.

#### Berberine (BBR)

BBR is an isoquinoline alkaloid derived mainly from Coptidis Rhizoma, which is officially listed in the Chinese Pharmacopoeia and commonly used for the treatment of diabetes 72. BBR was recently found to exert antineoplastic activity in HCC by inducing oxidative stress and enhancing radiosensitivity via suppressing Nrf2 signaling pathway in HCC cells. Later, BBR was also found to enhance the growth inhibitory effect of radiation in a Nrf2-dependent manner in a xenograft mouse model 73. Further studies also suggested that BBR could reverse lapatinib resistance of Her2-positive breast cancer cells by upregulating the level of ROS through inhibiting Nrf2 pathway 74. The above results have amply demonstrated that BBR is a promising sensitizer for chemotherapy.

### Quassinoid compounds

Quassinoids are a group of compounds extracted from plants of the Simaroubaceae family, which have been used for many years in folk medicine. Quassinoids have attracted increasing attention due to their promising biological activities, especially in relation to active anticancer principles 75. Among them, brusatol and brucein D (BD) are the most studied quassinoids, and have been identified as Nrf2 inhibitors with potent anti-tumor activities.

#### Brusatol

Brusatol, a quassinoid first isolated from the seeds of *Brucea javanica* (Yadanzi in Chinese), which is known to possess heat-clearing and detoxification, and anti-malarial effects in Chinese medicine practice. As a quassinoid compound, brusatol specifically suppresses Nrf2 signaling, and sensitizes a broad spectrum of cancer cells to anticancer agents 76, 77. Additionally, it has been identified as a Nrf2 inhibitor and inhibited the Nrf2 transcriptional signature to enhance the chemosensitivity to several chemotherapeutics 78. Brusatol was also evaluated as an experimental therapeutic for P-388 lymphocytic leukemia model, and the results showed that brusatol exhibited potent inhibitory effect on tumor metabolism and proliferation through interfering with protein synthesis 79. A follow-up study further indicated that treatment with brusatol could augment the sensitivity of anticancer drug cytarabine and daunorubicin to acute myeloid leukemia (AML) cells, and reduce its colony formation ability by downregulating the protein expression of Nrf2. Brusatol exerted minimal effects on early apoptosis in AML cells at high concentration 80. It has also been shown that brusatol sensitizes lung cancer cell A549 and xenografts to chemotherapeutic drugs, including carboplatin, etoposide, 5-fluorouracil and paclitaxel. The effects were found to be associated with downregulation of the protein expression of Nrf2 and accumulation of ROS, resulting in DNA damage and enhancement of radiation-sensitivity 67, 81.

Mechanistically, brusatol inhibits Nrf2 to overcome chemoresistance through inhibition of protein translation 82. Also, brusatol decreased A549 cell viability and induced apoptosis independent of caspase-3 via the inhibition of Nrf2 activity 83. Further research also showed that brusatol decreased the viability and sensitized CRC cells to irinotecan toxicity, and abrogated CRC tumor growth in subcutaneously and orthotopically-allografted mice through inhibiting Nrf2 84. Additionally, brusatol provokes a rapid and transient depletion of Nrf2 protein through a posttranscriptional mechanism in mouse Hepa-1c1c7 hepatoma cells and inhibits Nrf2 in freshly isolated primary human liver cancer cells 85. In another study conducted on melanoma A375 cells, co-treatment with brusatol and ultraviolet A resulted in an inhibition of A375 cell proliferation and reduction of melanoma-derived tumor burden, and the anticancer effects were through triggering ROS-dependent apoptosis and blocking AKT-Nrf2 pathway 86. Furthermore, brusatol demonstrated significantly inhibitory effects against breast cancer MCF-7 cells with IC_50_ 0.08 μM/L and improved the sensitivity of MCF-7 cells to Taxol via suppressing Nrf2 pathway and enhancing ROS levels of the breast cancer cells 87. Brusatol was also found to exert inhibitory effect on glioma, and its mechanisms were associated with the Nrf2 pathway blockage via suppressing glutathione synthesis and accumulating ROS, eventually leading to tumor cell damage 88. These findings have amply demonstrated that the inhibitory effect of brusatol on Nrf2 is associated with the global inhibition of protein synthesis, suggesting that Nrf2 signaling can be used as an anticancer therapeutic target, and brusatol as a naturally occurring potent Nrf2 inhibitor is worthy of further development into anticancer therapeutics.

#### Brucein D (BD)

BD, another quassinoid compound, was also first isolated from the seeds of *Brucea javanica.* This herbal plant has been used in Chinese medicine practice to treat inflammation, malaria, warts and cancers 89. BD has been demonstrated in a number of studies to exhibit excellent anticancer activities owing to its ability to inhibit proliferation, induce apoptosis and improve chemoresistance 89-91. For instance, BD was able to inhibit antiapoptotic activity elicited by NF-κB activation in pancreatic cancer cells 92, and BD treatment also induced pancreatic cell apoptosis through the activation of p38-mitogen activated protein kinase and mitochondrial pathways 93, 94. Besides, BD exerted anti-migratory activity by repressing PI3K/Akt signaling in breast cancer cells 95. Study has also shown that BD could effectively inhibit human pancreatic ductal adenocarcinoma (PDAC) cell proliferation, induce apoptosis, and enhance the chemosensitivity of gemcitabine in a genetically engineered Kras^tm4Tyj^ Trp53^tm1Brn^ Tg (Pdx1-cre/Esr1*) #Dam/J (KPC) mouse model through inhibiting Nrf2 expression via promoting the ubiquitin-proteasome-dependent degradation and downregulating its downstream genes such as HO-1, NQO1, AKR1B10 and γGCSm 96.

### Other promising compounds

Other natural compounds, such as cryptotanshinone (CTS), ginsenoside Rd (GS-Rd), ascorbic acid (AA), triptolide (TPL), have also been found to exert antineoplastic effects through suppressing Nrf2 activities.

#### Cryptotanshinone (CTS)

CTS, also named cryptotanshinone or tanshinone C, is a cell-permeable diterpene quinone. It is also one of the major tanshinones (including tanshinone I, tanshinone IIA, dihydrotanshinone and cryptotanshinone) derived from the root of *Salvia miltiorrhiza* (Danshen in Chinese). CTS has been demonstrated to be a potent antitumor agent due to its ability to induce cell death and apoptosis in human lung carcinoma A549 (A549/DDP) cells and enhance the sensitivity of cisplatin by down-regulating Nrf2 pathway 97.

#### Ginsenoside Rd (GS-Rd)

Ginsenosides are the major active ingredients of ginseng and display a wide variety of pharmacological effects, including antioxidant, anti-inflammatory, immunomodulatory, vascular protective, neuroprotective, and anticancer activities 98. GS-Rd, one of the principal active constituents of ginsenosides, is a potent antitumor agent 99. Studies conducted on non-small-cell lung cancer (NSCLC) A549 cells demonstrated that GS-Rd could inhibit the proliferation of NSCLC A549 cells in a dose and time-dependent manners, and renders cells more sensitive to anticancer drug cisplatin, with combination index values much smaller than 1.0, indicating a synergistic action. Collectively, treatment with GS-Rd led to the inhibition of A549 cells through a marked depletion of the expression of Nrf2 and its target genes, resulting in an enhanced sensitivity of A549 cells to cisplatin and amelioration of chemoresistance 100. Considering its effective medicinal use, GS-Rd and other ginseng compounds have good potential for combination therapy of NSCLC.

#### Ascorbic acid (AA)

AA, also known as vitamin C, is a powerful antioxidant and cofactor that participates in diverse enzymatic reactions, and has been shown to have anticancer property by selectively inducing ROS in cancer cells 101. AA suppresses oxidative stress to inhibit the nuclear translocation of Nrf2, and treatment with AA sensitizes cells to imatinib in imatinib-resistant chronic myelogenous leukemia KCL22/SR cells 102. However, in another study, AA was shown to induce the production of high levels of hydrogen peroxide, resulting in inhibition rather than activation of Nrf2 and HO-1 expression in Hug7 liver cancer cells 103. Thus, further work is needed to determine the involvement of Nrf2 pathway associated with the anticancer effect of AA.

#### Triptolide (TPL)

TPL is a diterpenoid epoxide originated from *Tripterygium wilfordii*. It has been used in Chinese medicine to treat autoimmune and inflammatory diseases, such as rheumatoid arthritis and lupus erythematosus, for a long time. Recent studies have demonstrated that TPL exhibits antitumor properties against various cancer types, such as breast, lung, and pancreatic cancer. Of significance, TPL has been found to increase the sensitivity of a drug-resistant human leukemia cell line to chemotherapeutic agents such as doxorubicin and imatinib by reducing the expression of Nrf2. Further studies identified that TPL significantly reduced Nrf2 expression and transcriptional activity in NSCLC and liver cancer cells, which resulted in chemosensitivity to antitumor drugs *in vitro* and in a xenograft tumor model system using lung carcinoma cells 104. Additionally, TPL was shown to induce cardiotoxicity through oxidative stress, downregulate Nrf2 activation, and the mitochondria-mediated apoptotic signaling pathway 105. However, inhibiting Nrf2 activity in combination with a neomorphic IDH1 mutation resulted in synergistic lethality. TPL was also found to be a potent Nrf2 inhibitor, and could induce apoptotic changes in IDH1-mutated cells through redox catastrophe 106. Above studies indicated that TPL could target the Nrf2 pathway, thereby preventing cancer progression. Further investigation is warranted to evaluate the efficacy and safety of TPL in clinical cancer trials.

Collectively, these data manifest that natural products and their derivatives might be promising anticancer agents. Although additional studies are required to better characterize the toxicity profile as well as the efficacy of these agents in specific clinical contexts, there is undisputable evidence that these compounds hold great promises in the treatment of malignant tumors, particularly in those cancer types with Nrf2 aberrant expression.

## Recent Insights in the Clinical Investigation of Natural Nrf2 Inhibitors

Nrf2 inhibitors are natural or synthetic compounds that are designed to target the Nrf2 pathway in order to exert therapeutic effects in various diseases, including cancer. Although no Nrf2 targeted therapy has been approved for cancer treatment, the results of some early-phase clinical trials on targeting Nrf2 have revealed promising results for cancer treatment. For instance, halofuginone is being tested in Phase I/II clinical trials for the treatment of AIDS-related Kaposi Sarcoma and recurrent Kaposi Sarcoma (NCT00027677 and NCT00064142, respectively). Other clinical trials have assessed the safety and antitumor activity of BBR in patients with colorectal, lung and gastric carcinoma (NCT03281096, NCT03486496, NCT04697186, NCT03609892, respectively). **Table 2** presents the Nrf2 inhibitors that have entered into clinical trials (http://clinicaltrials.gov/).

However, the safety of Nrf2 inhibitors is an area of ongoing research and investigation. Some studies have raised concerns about potential adverse effects of Nrf2 inhibitors, including liver toxicity, adverse cardiovascular effects, and potential interactions with other medications. Additionally, the safety and efficacy of any natural products or synthetic compounds can vary depending on the specific dose, route of administration, and individual patient factors. It is worth bearing in mind that the safety and efficacy of Nrf2 inhibitors need to be carefully evaluated before they can be used as cancer therapeutics.

## Discussion and Conclusions

Transcription factor Nrf2 constitutes an attractive drug target for the treatment of various cancers owing to its capacity of controlling the intracellular redox homeostasis and its ability to activate cytoprotective antioxidant genes. Hence, attention should be paid to the potential role of Nrf2/Keap1 pathway in interaction with anticancer drugs. Notably, natural compounds are well known for their multiple-targets, multi-pathways and relatively less toxic in cancer treatment. Nrf2 inhibitors have shown potential advantages as a cancer therapy, including sensitizing cancer cells to chemotherapy and overcoming drug resistance. The exact molecular mechanisms underlying these effects may vary depending on the specific inhibitor used and the type of cancer being targeted. For example, some Nrf2 inhibitors may work by directly binding to and blocking Nrf2 activity, while others may affect upstream regulators or downstream effectors of the pathway. Moreover, different cancer types may have unique dependencies on Nrf2, which can influence the therapeutic response to Nrf2 inhibition. Studies have shown that Nrf2 is overexpressed in a variety of cancer types, including lung, breast, ovarian, pancreatic, colorectal, and liver cancer. Therefore, Nrf2 blocking therapy may be more effective in treating these types of cancer. However, further research is needed to fully understand the molecular mechanisms of Nrf2 inhibitors in different cancers. Direct comparisons between different inhibitors and cancer types would be helpful in elucidating the specific mechanisms of action and identifying optimal treatment strategies.

This review has outlined the current state of Nrf2 inhibitors derived from natural sources and summarize their corresponding molecular mechanisms in regulating Nrf2 for cancer treatment. The natural compounds discussed in this review possess promising anticancer activity as evidenced by* in vitro* and *in vivo* experimental findings, and some of them have already been subjected to clinical trials. However, clinical research on selective Nrf2 inhibitors is still in its infancy, and some limitations to their use must be noted. One such limitation is their potential lack of specificity 107, since naturally occurring Nrf2 inhibitors may interact with multiple pathways. Therefore, Nrf2 inhibitors may possess a potential risk of unintended side effects or toxicity. The lack of specificity can give rise to difficulty in identifying the exact mechanism by which they exert their effects 108. Another limitation is the potency and bioavailability of natural product-derived Nrf2 inhibitors, which can vary widely. Some natural products may have low potency, while others may have limited bioavailability due to factors such as poor solubility and rapid metabolism. These issues can affect the efficacy of the Nrf2 inhibitor in a clinical setting 27. This can create challenges for regulatory approval and commercialization of natural product-derived Nrf2 inhibitors. These limitations need to be addressed before their safe and effective use in clinical settings.

Taken together, Nrf2 pathway is essential for tumorigenesis, tumor progression and resistance to therapy, and is clearly a promising target for cancer therapy, especially in cancers with high Nrf2 activity. The development of more specific and effective Nrf2 inhibitors will be necessary to improve the therapeutic potential of these drugs. Particularly, molecular docking model, a critical approach in drugs discovery and development, can be used to predict the binding affinity and orientation of ligands (natural inhibitors) with the target protein Nrf2 based on their 3D chemical structures 109. We anticipate that further in-depth studies such as biological and structure-activity relationship investigations on the natural Nrf2 inhibitors may eventually help develop naturally occurring Nrf2 inhibitors into anticancer therapeutics.

## Figures and Tables

**Table 1:** Anticancer effects of naturally occurring Nrf2 inhibitors and associated action mechanisms.

Active compounds	Cancer type	Model	Dose	Mechanisms of action	Ref.
Apigenin	HCC	HCC cells (BEL-7402) and BEL-7402/ADM cells	20 μM;50 mg/kg	↓Nrf2, ↓HO-1, AKR1B10, MRP5; ↓p-Akt	53
Luteolin	Lung cancer	A549 cells	1-20 μM;	↓Nrf2, ↓HO-1, NQO1, AKR1C	55
	CRC	HCT116 and SW620	10 μM; 40 mg/kg	↓Nrf2, ↓HO-1, NQO1, GSTα1/2	56
	Breast CSCs	MDA-MB-231	1-2 μM	↓Nrf2, ↓ABCG2, Nanog, Oct4, Cripto1, Sirt3, HO-1	57
Wogonin	CML	K562 cells	40 μM; 40 mg/kg	↓Nrf2, ↓P65, P50, p-Stat3, NF-κB pathway	59
	CML	K562 cells	40 μM;	↓Nrf2, ↓MRP1, NQO1, HO-1, PI3K, p-Akt,	60
Chrysin	Glioblastoma	T98, U251, U87 cells	30/60 μM; 40/80 mg/kg	↓Nrf2, ↓HO-1, NQO1, ↓p-JNK, p-P38, p-ERK	62
	HCC	BEL-7402 cells	10 μM	↓Nrf2, ↓HO-1, AKR1B10, MRP5; ↓p-Akt, p-JNK, p-ERK, p-P38	63
Halofuginone	Lung cancer	A549, KYSE70 and ABC1 cells	50-100 nM; 0.25 mg/kg	↓Nrf2, ↓NQO1	69
Trigonelline	Pancreatic cancer	MiaPaca2, Panc1 and Colo357 cells	0.1/0.5 μM; 0.02 mg/kg	↓Nrf2	71
Berberine	HCC	Huh7, HepG2 and HHL-5 cells	20 μM; 5 mg/kg	↓Nrf2, ↓HO-1, NQO1	73
	Breast cancer	BT-474 and AU-565 cells	2 μM; 5 mg/kg	↓Nrf2, ↓p-GSK3β, C-Myc	74
Brusatol	AML	THP1, Molm13, U937, HL60 cells	1 μM	↓Nrf2	80
	Lung cancer	A549 cells	40 nM; 2 mg/kg	↓Nrf2, ↑Keap-1 ↓MRP1, MRP2, NQO1, γ-GCS,	78
		A549 cells	40 nM	↓Nrf2, ↓NQO1, GCLC, GCLM	83
	CRC	HCT116, CT26	300 nM; 2 mg/kg	↓Nrf2, ↑Keap-1	84
	Hepatoma	Mouse Hepa-1c1c7 cells	300 nM;	↓Nrf2, ↓NQO1	85
	Melanoma	A357 cells	100 nM; 2 mg/kg	↓Nrf2, ↓HO-1, NQO1, GSTP1	86
	Breast cancer	MCF-7, MDA-MB-231	40 nM	↓Nrf2, ↑Keap-1, ↓NQO1, GCLM, HO-1	87
	Glioma	U-251	2 ng/mL	↓Nrf2, ↓NQO1, GCLC	88
Brucein D	PDAC	Miapaca-2, Capan2 and PANC1 cells	1.5 μM; 2 mg/kg	↓Nrf2, ↓HO-1, NQO1, AKR1B10 γGCSm, MRP1 and MRP5	96
Cryptotanshinone	Lung cancer	A549 cells	10 μM	↓Nrf2, ↓MRP1, GCLC, GCLM, HO-1, NQO1, ↓p-Akt, p-STAT3, p-JNK, p-ERK	97
Ginsenoside Rd	NSCLC	A549 cells	80 μM	↓Nrf2, ↓HO-1, NQO1, GCLC, MRP1,	100
Ascorbic acid	CML	KCL22 cells	0.125 mM	↓Nrf2	102
Triptolide	NSCLC/liver cancer	A549 cells and HepG2	0.5 μM; 0.25 mg/kg	↓Nrf2	104
	Heart	H9c2 cells	1.2 mg/kg	↓Nrf2, ↓Bax/Bcl-2, ↑Caspase 3	105
	Glioblastoma	U251 MG cells	30 nM	↓Nrf2, ↓GCLC, GCLM	106

**Table 2:** Overview of the clinical trials on natural Nrf2 inhibitors for cancer treatment.

Compounds	Study design	Intervention/treatment	Tumor types	Phase	Status	Primary endpoint	Clinical trial identifier
Apigenin	17 participants, Non-Randomized, Crossover Assignment, Open label	Chamomile Tea	Health	N/A	Completed	Metabolites in plasma/urine	NCT03526081
Luteolin	4 participants, Non-Randomized, Parallel Assignment,	Luteolin, nano-luteolin	Tongue Neoplasms Carcinoma	Early I	N/R	gene expression of Caspase 3 to detect apoptosis	NCT03288298
Wogonin	/	/	/	/	N/R	/	N/A
Chrysin	/	/	/	/	N/R	/	N/A
Halofuginone	25 participants	halofuginone hydrobromide	Unspecified Adult Solid Tumor, Protocol Specific	I	Completed	N/R	NCT00027677
	30 participants, Randomized, Parallel Assignment,	halofuginone hydrobromide , placebo	AIDS-related Kaposi Sarcoma, Recurrent Kaposi Sarcoma	II	Completed	Safety, RR	NCT00064142
Trigonelline	/	/	/	/	N/R	/	N/A
Berberine	1000 participants, Randomized, Parallel Assignment,	Berberine hydrochloride, placebo	Colorectal Adenomas	II-III	Completed	Incidence rate	NCT03281096
	50 participants, Single Group Assignment, open label	Gefitinib, berberine	Lung Adenocarcinoma EGFR Mutation	II	N/R	PFS; Safety	NCT03486496
	18 participants, Randomized, Parallel Assignment	Berberine chloride, laboratory biomarker analysis, placebo administration	Ulcerative Colitis	I	Completed	Safety	NCT02365480
	524 participants, Randomized, Parallel Assignment	Berberine, amoxicillin, rabeprazole, clarithromycin, bismuth	Gastric Cancer	IV	Enrolling by invitation	Helicobacter pylori, symptoms effective rate	NCT04697186
	658 participants, Randomized, Parallel Assignment	Berberine, amoxicillin, esomeprazole bismuth, etracycline, furazolidone	Gastric Cancer	IV	Completed	Helicobacter pylori, symptoms effective rate	NCT03609892
	1108 participants, Randomized, Parallel Assignment	Berberine hydrochloride, placebo	Colorectal Adenoma	II-III	Completed	Recurrence rates	NCT02226185
	100 participants, Randomized, Parallel Assignment	Berberine hydrochloride	Colorectal Adenomas	II-III	Completed	Measured tumor	NCT03333265
Brusatol	/	/	/	/	N/R	/	N/A
Brucein D	/	/	/	/	N/R	/	N/A
Cryptotanshinone	/	/	/	/	N/R	/	N/A
Ginsenoside Rd	/	/	/	/	N/R	/	N/A
Ascorbic acid	30 participants, Randomized, Parallel Assignment	Ascorbic acid	Breast Cancer	I-II	N/R	Safety, tolerability and RR	NCT03175341
	21 participants, Single Group Assignment, open label	Ascorbic acid	Bladder Cancer	I-II	Recruiting	Pathological Staging	NCT04046094
	200 participants, Randomized, Parallel Assignment	Ascorbic acid in combination with mFOLFOX6	Gastric Cancer	III	N/R	PFS, OS, RR	NCT03015675
	31 participants, Single Group Assignment, open label	Vitamin C	Prostatic Neoplasms	II	Completed	Biomarkers change (PSA, bALP, NTX, PINP)	NCT01080352
	15 participants, Single Group Assignment, open label	Gemcitabine with escalating ascorbic acid	Pancreatic Neoplasms	I	Completed	Blood cell counts	NCT01049880
	97 participants, Randomized, Parallel Assignment	Vitamin C + supportive care	Stage IIIA-B, IV NSCLC Recurrent NSCLC	I-II	Completed	Safety, tolerability, anti-tumor activity, PFS, OS	NCT02655913
	5 participants, Non-Randomized, Parallel Assignment	Ascorbic acid in combination with sorafenib	Metastatic Hepatocellular Carcinoma Advanced Liver Cancer	I-II	Completed	Safety, overall tumor response rate	NCT01754987
	60 participants, Randomized, Parallel Assignment	Vitamin C, E and Zinc	Skin Neoplasms	N/R	Completed	Oxidative stress biomaker	NCT02248584
	42 participants, Randomized, Parallel Assignment	Nutritional supplement	Head and Neck Cancer	N/R	Completed	POSAS	NCT03531190
Triptolide	30 participants, Single Group Assignment, open label	Procedure: Biopsy; osimertinib, triptolide analog	Advanced lung non-small cell carcinoma	I	Recruiting	MTD, PFS, OS	NCT05166616
	66 participants, Non-Randomized, Parallel Assignment,	Minnelide™Capsules	Gastric, breast, pancreatic, prostate, colorectal cancer, solid tumor, solid carcinoma of stomach, cancer of stomach	I	Recruiting	Safety, anti-tumor activity	NCT03129139
	55 participants, Single Group Assignment, open label	Minnelide	Adenosquamous carcinoma of the pancreas	II	Recruiting	DCR, PFS, safety, tolerability, OS	NCT04896073
	19 participants, Single Group Assignment, open label	Minnelide	Pancreatic cancer	II	Completed	DCR, PFS, OS, RR	NCT03117920

The list above did not include those studies that were either suspended or terminated prematurely. (N/A, not applicable; N/R, not reported; NSCLC, non-small cell lung cancer; POSAS, The patient and observer scar assessment scale; MTD, Maximum tolerated dose; PFS, Progression-free survival; OS, Overall survival; DCR, Disease control rate; RR, Response rate).

## Abbreviations

AA: ascorbic acid
ADM: doxorubicin
ADR: adriamycin
APG: Apigenin
ARE: antioxidant-responsive element
AML: acute myeloid leukemia
BBR: berberine
BD: brucein D
b-Zip: basic leucine Zipper domain
CHM: Chinese herbal medicine
CML: chronic myelogenous leukemia
CRC: colorectal cancer
CSCs: cancer stem cells
CTS: cryptotanshinone
CUL3: Cullin 3
EMT: epithelial-mesenchymal transition
GS-Rd: ginsenoside Rd
HCC: hepatocellular carcinoma
HO-1: hemeoxygenase-1
Keap1: KELCH-like ECH-associated protein 1
Neh: Nrf2-ECH homology
Nrf2: Nuclear factor erythroid 2-related factor 2
NSCLC: non-small-cell lung cancer
PDAC: pancreatic ductal adenocarcinoma
PI3K: phosphatidylinositol-3 kinase
ROS: reactive oxygen species
sMafs: small musculoaponeurotic fibrosarcoma proteins
TPL: triptolide
TRAIL: tumor necrosis factor-related apoptosis inducing ligand

## References

[1] George BP, Chandran R, Abrahamse H (2021). Role of Phytochemicals in Cancer Chemoprevention: Insights. Antioxidants (Basel).

[2] Sung H, Ferlay J, Siegel RL, Laversanne M, Soerjomataram I, Jemal A (2021). Global Cancer Statistics 2020: GLOBOCAN Estimates of Incidence and Mortality Worldwide for 36 Cancers in 185 Countries. CA Cancer J Clin.

[3] Hanahan D, Weinberg RA (2011). Hallmarks of cancer: the next generation. Cell.

[4] Radisky DC, Levy DD, Littlepage LE, Liu H, Nelson CM, Fata JE (2005). Rac1b and reactive oxygen species mediate MMP-3-induced EMT and genomic instability. Nature.

[5] Nishikawa M (2008). Reactive oxygen species in tumor metastasis. Cancer Lett.

[6] Perillo B, Di Donato M, Pezone A, Di Zazzo E, Giovannelli P, Galasso G (2020). ROS in cancer therapy: the bright side of the moon. Exp Mol Med.

[7] Gorrini C, Harris IS, Mak TW (2013). Modulation of oxidative stress as an anticancer strategy. Nat Rev Drug Discov.

[8] Hornsveld M, Dansen TB (2016). The Hallmarks of Cancer from a Redox Perspective. Antioxid Redox Signal.

[9] Marengo B, Nitti M, Furfaro AL, Colla R, Ciucis CD, Marinari UM (2016). Redox Homeostasis and Cellular Antioxidant Systems: Crucial Players in Cancer Growth and Therapy. Oxid Med Cell Longev.

[10] Kansanen E, Kuosmanen SM, Leinonen H, Levonen AL (2013). The Keap1-Nrf2 pathway: Mechanisms of activation and dysregulation in cancer. Redox Biol.

[11] Taguchi K, Motohashi H, Yamamoto M (2011). Molecular mechanisms of the Keap1-Nrf2 pathway in stress response and cancer evolution. Genes Cells.

[12] Sova M, Saso L (2018). Design and development of Nrf2 modulators for cancer chemoprevention and therapy: a review. Drug Des Devel Ther.

[13] de la Vega MR, Chapman E, Zhang DD (2018). NRF2 and the Hallmarks of Cancer. Cancer Cell.

[14] Panieri E, Saso L (2019). Potential Applications of NRF2 Inhibitors in Cancer Therapy. Oxidative Medicine and Cellular Longevity. 2019.

[15] Taguchi K, Yamamoto M (2017). The KEAP1-NRF2 System in Cancer. Frontiers in Oncology.

[16] Wu KJ, Huang JM, Zhong HJ, Dong ZZ, Vellaisamy K, Lu JJ (2017). A natural product-like JAK2/STAT3 inhibitor induces apoptosis of malignant melanoma cells. PLoS One.

[17] Alia M, Ramos S, Mateos R, Granado-Serrano AB, Bravo L, Goya L (2006). Quercetin protects human hepatoma HepG2 against oxidative stress induced by tert-butyl hydroperoxide. Toxicol Appl Pharm.

[18] Farombi EO, Shrotriya S, Na HK, Kim SH, Surh YJ (2008). Curcumin attenuates dimethylnitrosamine-induced liver injury in rats through Nrf2-mediated induction of heme oxygenase-1. Food Chem Toxicol.

[19] Granado-Serrano AB, Martin MA, Bravo L, Goya L, Ramos S (2012). Quercetin modulates Nrf2 and glutathione-related defenses in HepG2 cells: Involvement of p38. Chem-Biol Interact.

[20] Lin W, Wu RT, Wu TY, Khor TO, Wang H, Kong AN (2008). Sulforaphane suppressed LPS-induced inflammation in mouse peritoneal macrophages through Nrf2 dependent pathway. Biochem Pharmacol.

[21] Talalay P, Fahey JW (2001). Phytochemicals from cruciferous plants protect against cancer by modulating carcinogen metabolism. Journal of Nutrition.

[22] Wu LY, Ashraf MHN, Facci M, Wang R, Paterson PG, Ferrie A (2004). Dietary approach to attenuate oxidative stress, hypertension, and inflammation in the cardiovascular system. P Natl Acad Sci USA.

[23] Kumar H, Kim IS, More SV, Kim BW, Choi DK (2014). Natural product-derived pharmacological modulators of Nrf2/ARE pathway for chronic diseases. Nat Prod Rep.

[24] Jaramillo MC, Zhang DD (2013). The emerging role of the Nrf2-Keap1 signaling pathway in cancer. Genes Dev.

[25] Smolkova K, Miko E, Kovacs T, Leguina-Ruzzi A, Sipos A, Bai P (2020). Nuclear Factor Erythroid 2-Related Factor 2 in Regulating Cancer Metabolism. Antioxid Redox Signal.

[26] Sanchez-Ortega M, Carrera AC, Garrido A (2021). Role of NRF2 in Lung Cancer. Cells.

[27] Wu S, Lu H, Bai Y (2019). Nrf2 in cancers: A double-edged sword. Cancer Med.

[28] Kim J, Keum YS (2016). NRF2, a Key Regulator of Antioxidants with Two Faces towards Cancer. Oxidative Medicine and Cellular Longevity. 2016.

[29] Fukutomi T, Takagi K, Mizushima T, Ohuchi N, Yamamoto M (2014). Kinetic, Thermodynamic, and Structural Characterizations of the Association between Nrf2-DLGex Degron and Keap1. Mol Cell Biol.

[30] Ooi BK, Chan KG, Goh BH, Yap WH (2018). The Role of Natural Products in Targeting Cardiovascular Diseases via Nrf2 Pathway: Novel Molecular Mechanisms and Therapeutic Approaches. Front Pharmacol.

[31] Itoh K, Chiba T, Takahashi S, Ishii T, Igarashi K, Katoh Y (1997). An Nrf2 small Maf heterodimer mediates the induction of phase II detoxifying enzyme genes through antioxidant response elements. Biochem Bioph Res Co.

[32] Rushmore TH, Pickett CB (1990). Transcriptional Regulation of the Rat Glutathione S-Transferase-Ya Subunit Gene - Characterization of a Xenobiotic-Responsive Element Controlling Inducible Expression by Phenolic Antioxidants. J Biol Chem.

[33] Zhu M, Fahl WE (2001). Functional characterization of transcription regulators that interact with the electrophile response element. Biochem Bioph Res Co.

[34] Li XC, He P, Wang XL, Zhang SN, Devejian N, Bennett E (2018). Sulfiredoxin-1 enhances cardiac progenitor cell survival against oxidative stress via the upregulation of the ERK/NRF2 signal pathway. Free Radical Bio Med.

[35] Li CJ, Cheng L, Wu HT, He PJ, Zhang YP, Yang Y (2018). Activation of the KEAP1-NRF2-ARE signaling pathway reduces oxidative stress in Hep2 cells. Molecular Medicine Reports.

[36] Kitano Y, Baba Y, Nakagawa S, Miyake K, Iwatsuki M, Ishimoto T (2018). Nrf2 promotes oesophageal cancer cell proliferation via metabolic reprogramming and detoxification of reactive oxygen species. J Pathol.

[37] De Nicola GM, Karreth FA, Humpton TJ, Gopinathan A, Wei C, Frese K (2011). Oncogene-induced Nrf2 transcription promotes ROS detoxification and tumorigenesis. Nature.

[38] Wu SJ, Lu H, Bai YH (2019). Nrf2 in cancers: A double-edged sword. Cancer Med-Us.

[39] Enomoto A, Itoh K, Nagayoshi E, Haruta J, Kimura T, O'Connor T (2001). High sensitivity of Nrf2 knockout mice to acetaminophen hepatotoxicity associated with decreased expression of ARE-regulated drug metabolizing enzymes and antioxidant genes. Toxicol Sci.

[40] Yang Y, Karakhanova S, Werner J, Bazhin AV (2013). Reactive oxygen species in cancer biology and anticancer therapy. Curr Med Chem.

[41] Rojo de la Vega M, Chapman E, Zhang DD (2018). NRF2 and the Hallmarks of Cancer. Cancer Cell.

[42] Padmanabhan B, Tong KI, Ohta T, Nakamura Y, Scharlock M, Ohtsuji M (2006). Structural basis for defects of Keap1 activity provoked by its point mutations in lung cancer. Mol Cell.

[43] Choi BH, Kim JM, Kwak MK (2021). The multifaceted role of NRF2 in cancer progression and cancer stem cells maintenance. Arch Pharm Res.

[44] Kaspar JW, Niture SK, Jaiswal AK (2009). Nrf2:INrf2 (Keap1) signaling in oxidative stress. Free Radical Bio Med.

[45] Kitamura H, Motohashi H (2018). NRF2 addiction in cancer cells. Cancer Sci.

[46] Sporn MB, Liby KT (2012). NRF2 and cancer: the good, the bad and the importance of context. Nat Rev Cancer.

[47] Shibata T, Ohta T, Tong KI, Kokubu A, Odogawa R, Tsuta K (2008). Cancer related mutations in NRF2 impair its recognition by Keap1-Cul3 E3 ligase and promote malignancy. Proc Natl Acad Sci U S A.

[48] Liu Y, Lang F, Yang C (2021). NRF2 in human neoplasm: Cancer biology and potential therapeutic target. Pharmacol Ther.

[49] Wang XJ, Sun Z, Villeneuve NF, Zhang S, Zhao F, Li YJ (2008). Nrf2 enhances resistance of cancer cells to chemotherapeutic drugs, the dark side of Nrf2. Carcinogenesis.

[50] Kopustinskiene DM, Jakstas V, Savickas A, Bernatoniene J (2020). Flavonoids as Anticancer Agents. Nutrients.

[51] Slika H, Mansour H, Wehbe N, Nasser SA, Iratni R, Nasrallah G (2022). Therapeutic potential of flavonoids in cancer: ROS-mediated mechanisms. Biomedicine & Pharmacotherapy.

[52] Javed Z, Sadia H, Iqbal MJ, Shamas S, Malik K, Ahmed R (2021). Apigenin role as cell-signaling pathways modulator: implications in cancer prevention and treatment. Cancer Cell Int.

[53] Gao AM, Ke ZP, Wang JN, Yang JY, Chen SY, Chen H (2013). Apigenin sensitizes doxorubicin-resistant hepatocellular carcinoma BEL-7402/ADM cells to doxorubicin via inhibiting PI3K/Akt/Nrf2 pathway. Carcinogenesis.

[54] No JH, Kim YB, Song YS (2014). Targeting nrf2 signaling to combat chemoresistance. J Cancer Prev.

[55] Tang XW, Wang HY, Fan LF, Wu XY, Xin A, Ren HY (2011). Luteolin inhibits Nrf2 leading to negative regulation of the Nrf2/ARE pathway and sensitization of human lung carcinoma A549 cells to therapeutic drugs. Free Radical Bio Med.

[56] Chian S, Li YY, Wang XJ, Tang XW (2014). Luteolin sensitizes two oxaliplatin-resistant colorectal cancer cell lines to chemotherapeutic drugs via inhibition of the Nrf2 pathway. Asian Pac J Cancer Prev.

[57] Tsai KJ, Tsai HY, Tsai CC, Chen TY, Hsieh TH, Chen CL (2021). Luteolin Inhibits Breast Cancer Stemness and Enhances Chemosensitivity through the Nrf2-Mediated Pathway. Molecules.

[58] Tai MC, Tsang SY, Chang LY, Xue H (2005). Therapeutic potential of wogonin: a naturally occurring flavonoid. CNS Drug Rev.

[59] Xu X, Zhang X, Zhang Y, Yang L, Liu Y, Huang S (2017). Wogonin reversed resistant human myelogenous leukemia cells via inhibiting Nrf2 signaling by Stat3/NF-kappaB inactivation. Sci Rep.

[60] Xu XF, Zhang Y, Li W, Miao HC, Zhang HW, Zhou YX (2014). Wogonin reverses multi-drug resistance of human myelogenous leukemia K562/A02 cells via downregulation of MRP1 expression by inhibiting Nrf2/ARE signaling pathway. Biochem Pharmacol.

[61] Xu D, Jin JZ, Yu H, Zhao ZM, Ma DY, Zhang CD (2017). Chrysin inhibited tumor glycolysis and induced apoptosis in hepatocellular carcinoma by targeting hexokinase-2. Journal of Experimental & Clinical Cancer Research.

[62] Wang J, Wang HD, Sun KJ, Wang XL, Pan H, Zhu JH (2018). Chrysin suppresses proliferation, migration, and invasion in glioblastoma cell lines via mediating the ERK/Nrf2 signaling pathway. Drug Des Dev Ther.

[63] Gao AM, Ke ZP, Shi F, Sun GC, Chen H (2013). Chrysin enhances sensitivity of BEL-7402/ADM cells to doxorubicin by suppressing PI3K/Akt/Nrf2 and ERK/Nrf2 pathway. Chem-Biol Interact.

[64] Lu JJ, Bao JL, Chen XP, Huang M, Wang YT (2012). Alkaloids Isolated from Natural Herbs as the Anticancer Agents. Evid-Based Compl Alt. 2012.

[65] Mondal A, Gandhi A, Fimognari C, Atanasov AG, Bishayee A (2019). Alkaloids for cancer prevention and therapy: Current progress and future perspectives. European Journal of Pharmacology.

[66] Keller TL, Zocco D, Sundrud MS, Hendrick M, Edenius M, Yum J (2012). Halofuginone and other febrifugine derivatives inhibit prolyl-tRNA synthetase. Nat Chem Biol.

[67] Lin HZ, Qiao YT, Yang HY, Nan Q, Qu W, Feng F (2020). Small molecular Nrf2 inhibitors as chemosensitizers for cancer therapy. Future Med Chem.

[68] Naciri M, Mancassola R, Yvore P, Peeters JE (1993). The effect of halofuginone lactate on experimental Cryptosporidium parvum infections in calves. Vet Parasitol.

[69] Tsuchida K, Tsujita T, Hayashi M, Ojima A, Keleku-Lukwete N, Katsuoka F (2017). Halofuginone enhances the chemo-sensitivity of cancer cells by suppressing NRF2 accumulation. Free Radic Biol Med.

[70] Boettler U, Sommerfeld K, Volz N, Pahlke G, Teller N, Somoza V (2011). Coffee constituents as modulators of Nrf2 nuclear translocation and ARE (EpRE)-dependent gene expression. J Nutr Biochem.

[71] Arlt A, Sebens S, Krebs S, Geismann C, Grossmann M, Kruse ML (2013). Inhibition of the Nrf2 transcription factor by the alkaloid trigonelline renders pancreatic cancer cells more susceptible to apoptosis through decreased proteasomal gene expression and proteasome activity. Oncogene.

[72] Dou Y, Huang R, Li Q, Liu Y, Li Y, Chen H (2021). Oxyberberine, an absorbed metabolite of berberine, possess superior hypoglycemic effect via regulating the PI3K/Akt and Nrf2 signaling pathways. Biomed Pharmacother.

[73] You X, Cao X, Lin Y (2019). Berberine enhances the radiosensitivity of hepatoma cells by Nrf2 pathway. Front Biosci (Landmark Ed).

[74] Zhang R, Qiao H, Chen S, Chen X, Dou K, Wei L (2016). Berberine reverses lapatinib resistance of HER2-positive breast cancer cells by increasing the level of ROS. Cancer Biol Ther.

[75] Houël E, Stien D, Bourdy G, Deharo E (2013). Quassinoids: Anticancer and Antimalarial Activities. In: Ramawat KG, Mérillon J-M, editors. Natural Products: Phytochemistry, Botany and Metabolism of Alkaloids, Phenolics and Terpenes. Berlin, Heidelberg: Springer Berlin Heidelberg.

[76] Yu XQ, Shang XY, Huang XX, Yao GD, Song SJ (2020). Brusatol: A potential anti-tumor quassinoid from Brucea javanica. Chin Herb Med.

[77] Lee JH, Mohan CD, Deivasigamani A, Jung YY, Rangappa S, Basappa S (2020). Brusatol suppresses STAT3-driven metastasis by downregulating epithelial-mesenchymal transition in hepatocellular carcinoma. J Adv Res.

[78] Ren D, Villeneuve NF, Jiang T, Wu T, Lau A, Toppin HA (2011). Brusatol enhances the efficacy of chemotherapy by inhibiting the Nrf2-mediated defense mechanism. Proc Natl Acad Sci U S A.

[79] Cai SJ, Liu Y, Han S, Yang C (2019). Brusatol, an NRF2 inhibitor for future cancer therapeutic. Cell Biosci.

[80] Karathedath S, Rajamani BM, Aalam SMM, Abraham A, Varatharajan S, Krishnamurthy P (2017). Role of NF-E2 related factor 2 (Nrf2) on chemotherapy resistance in acute myeloid leukemia (AML) and the effect of pharmacological inhibition of Nrf2. Plos One.

[81] Sun X, Wang Q, Wang Y, Du L, Xu C, Liu Q (2016). Brusatol Enhances the Radiosensitivity of A549 Cells by Promoting ROS Production and Enhancing DNA Damage. Int J Mol Sci.

[82] Harder B, Tian W, La Clair JJ, Tan AC, Ooi A, Chapman E (2017). Brusatol overcomes chemoresistance through inhibition of protein translation. Mol Carcinogen.

[83] Li Y, Zhou Y, Ni HM, Zhong H, Ding WX (2016). Nrf2 But Not Autophagy Activation Is Associated with Resistance to EGFR Inhibitor-Induced Lung Tumor Cell Apoptosis. Faseb J.

[84] Evans JP WB, Sutton PA (2018). The Nrf2 inhibitor brusatol is a potent antitumour agent in an orthotopic mouse model of colorectal cancer. Oncotarget.

[85] Olayanju A, Copple IM, Bryan HK, Edge GT, Sison RL, Wong MW (2015). Brusatol provokes a rapid and transient inhibition of Nrf2 signaling and sensitizes mammalian cells to chemical toxicity-implications for therapeutic targeting of Nrf2. Free Radic Biol Med.

[86] Wang M, Shi GW, Bian CX, Nisar MF, Guo YY, Wu Y (2018). UVA Irradiation Enhances Brusatol-Mediated Inhibition of Melanoma Growth by Downregulation of the Nrf2-Mediated Antioxidant Response. Oxid Med Cell Longev. 2018.

[87] Wu T, Harder BG, Wong PK, Lang JE, Zhang DD (2015). Oxidative stress, mammospheres and Nrf2-new implication for breast cancer therapy?. Mol Carcinog.

[88] Liu Y, Lu Y, Celiku O, Li A, Wu Q, Zhou Y (2019). Targeting IDH1-Mutated Malignancies with NRF2 Blockade. J Natl Cancer Inst.

[89] Fan J, Ren D, Wang J, Liu X, Zhang H, Wu M (2020). Bruceine D induces lung cancer cell apoptosis and autophagy via the ROS/MAPK signaling pathway *in vitro* and *in vivo*. Cell Death Dis.

[90] Mohan CD, Liew YY, Jung YY, Rangappa S, Preetham HD, Chinnathambi A (2021). Brucein D modulates MAPK signaling cascade to exert multi-faceted anti-neoplastic actions against breast cancer cells. Biochimie.

[91] Lai ZQ, Ip SP, Liao HJ, Lu Z, Xie JH, Su ZR (2017). Brucein D, a Naturally Occurring Tetracyclic Triterpene Quassinoid, Induces Apoptosis in Pancreatic Cancer through ROS-Associated PI3K/Akt Signaling Pathway. Front Pharmacol.

[92] Lau ST, Lin ZX, Leung PS (2010). Role of reactive oxygen species in brucein D-mediated p38-mitogen-activated protein kinase and nuclear factor-kappaB signalling pathways in human pancreatic adenocarcinoma cells. Br J Cancer.

[93] Lau ST, Lin ZX, Liao YH, Zhao M, Cheng CHK, Leung PS (2009). Brucein D induces apoptosis in pancreatic adenocarcinoma cell line PANC-1 through the activation of p38-mitogen activated protein kinase. Cancer Lett.

[94] Liu L, Lin ZX, Leung PS, Chen LH, Zhao M, Liang J (2012). Involvement of the mitochondrial pathway in bruceine D-induced apoptosis in Capan-2 human pancreatic adenocarcinoma cells. Int J Mol Med.

[95] Luo C, Wang Y, Wei C, Chen YX, Ji ZN (2020). The anti-migration and anti-invasion effects of Bruceine D in human triple-negative breast cancer MDA-MB-231 cells. Exp Ther Med.

[96] Zhang J, Xu HX, Cho WCS, Cheuk W, Li Y, Huang QH (2022). Brucein D augments the chemosensitivity of gemcitabine in pancreatic cancer via inhibiting the Nrf2 pathway. J Exp Clin Cancer Res.

[97] Xia C, Bai XP, Hou XY, Gou XL, Wang YT, Zeng H (2015). Cryptotanshinone Reverses Cisplatin Resistance of Human Lung Carcinoma A549 Cells through Down-Regulating Nrf2 Pathway. Cell Physiol Biochem.

[98] Kim JH (2018). Pharmacological and medical applications of Panax ginseng and ginsenosides: a review for use in cardiovascular diseases. J Ginseng Res.

[99] Cho K, Song SB, Tung NH, Kim KE, Kim YH (2014). Inhibition of TNF-alpha-Mediated NF-kappaB Transcriptional Activity by Dammarane-Type Ginsenosides from Steamed Flower Buds of Panax ginseng in HepG2 and SK-Hep1 Cells. Biomol Ther (Seoul).

[100] Chian S, Zhao YN, Xu M, Yu XL, Ke X, Gao RL (2019). Ginsenoside Rd reverses cisplatin resistance in non-small-cell lung cancer A549 cells by downregulating the nuclear factor erythroid 2-related factor 2 pathway. Anti-Cancer Drug.

[101] Cho S, Chae JS, Shin H, Shin Y, Song H, Kim Y (2018). Hormetic dose response to (L)-ascorbic acid as an anti-cancer drug in colorectal cancer cell lines according to SVCT-2 expression. Sci Rep-Uk.

[102] Tarumoto T, Nagai T, Ohmine K, Miyoshi T, Nakamura M, Kondo T (2004). Ascorbic acid restores sensitivity to imatinib via suppression of Nrf2-dependent gene expression in the imatinib-resistant cell line. Exp Hematol.

[103] Wagner AE, Boesch-Saadatmandi C, Breckwoldt D, Schrader C, Schmelzer C, Doring F (2011). Ascorbic acid partly antagonizes resveratrol mediated heme oxygenase-1 but not paraoxonase-1 induction in cultured hepatocytes - role of the redox-regulated transcription factor Nrf2. BMC Complement Altern Med.

[104] Zhu J, Wang H, Chen F, Lv H, Xu Z, Fu J (2018). Triptolide enhances chemotherapeutic efficacy of antitumor drugs in non-small-cell lung cancer cells by inhibiting Nrf2-ARE activity. Toxicol Appl Pharmacol.

[105] Zhou J, Xi C, Wang WW, Fu XL, Jinqiang L, Qiu YW (2014). Triptolide-induced oxidative stress involved with Nrf2 contribute to cardiomyocyte apoptosis through mitochondrial dependent pathways. Toxicol Lett.

[106] Yu D, Liu Y, Zhou YQ, Ruiz-Rodado V, Larion M, Xu GW (2020). Triptolide suppresses IDH1-mutated malignancy via Nrf2-driven glutathione metabolism. P Natl Acad Sci USA.

[107] Panieri E, Buha A, Telkoparan-Akillilar P, Cevik D, Kouretas D, Veskoukis A (2020). Potential Applications of NRF2 Modulators in Cancer Therapy. Antioxidants (Basel).

[108] Srivastava R, Fernandez-Gines R, Encinar JA, Cuadrado A, Wells G (2022). The current status and future prospects for therapeutic targeting of KEAP1-NRF2 and beta-TrCP-NRF2 interactions in cancer chemoresistance. Free Radic Biol Med.

[109] Pinzi L, Rastelli G (2019). Molecular Docking: Shifting Paradigms in Drug Discovery. Int J Mol Sci.

